# Apparent Want of Action in Various Arteries

**Published:** 1817-06

**Authors:** 


### *Apparent Want of Action in various Arteries,* observed by Dr. Lara and Dr. Johnson.

An old superannuated soldier, 65 years of age, corpulent, dropsical, and afflicted with ulcerated legs, applied for gratuitous relief on the 10th of January of the present year. There was evident fluctuation in the abdomen; the urine was scanty, and the legs œdematous. There was a peculiarity in the countenance, which induced the Reporters to examine the region of the heart. No motion of that organ was perceptible in any position. The radial arteries were then felt for; but no pulse could be distinguished by either of the medical gentlemen present. The temporal artery on the left side was seen large and tortuous; but, on compressing it, no pulsation whatever was perceptible by either of the medical gentlemen. The other temporal artery also was destitute of pulsation. The carotids gave an obscure sensation of pulse. On being questioned if he felt different from what he had done for some days past, he replied in the negative. His health was bad, but not worse than usual. One of the medical gentlemen returned early the next morning to examine the patient, but found him in precisely the same state as to the arterial system. Some blue pill, aloes, squill, and digitalis, had been prescribed the preceding day. On the third morning one of the medical gentlemen returned. The patient said he had made a good deal of water, that his bowels were open, and that he felt much better. The pulsations were now perceptible in both radials and temporals. In the course of a week, the medicine seemed to lose its effect, and the urine to become again scanty. There was, however, an oozing of at least a pint of water daily from the ulcerated and œdematous legs. There was now evidently a great collection of water in the abdomen, and the patient informed Drs. Lara and Johnson, that he had once or twice lately felt some strange sensations in his chest, as though he were going to die. Great palpitation and anxiety in the region of the heart were felt at these times. On Wednesday, the 15th of January, the above-mentioned gentlemen visited the patient. They found him copying music, he having formerly belonged to the marine band. He said he found himself but indifferent. His urine was scanty, the abdomen much swelled, and still that indescribable expression of countenance which first attracted so much attention. All the arteries, however, were in full play, and distinctly pulsating. On the evening of this day, the nurse observed that the ulcers of the legs were suddenly dried up. He went to bed, at the usual hour, without complaining of any thing in particular, but was found dead next morning.

The singularity of this case, in respect to non-pulsation of the arteries for such a long space of time, excited much curiosity to see the state of the heart; and with some difficulty, permission was procured to examine the thorax.

*Sectio Cadaveris, thirty hours after death.* The face and neck quite livid with extravasated blood. The abdomen was an immense size with water, and anasarca prevailed pretty generally. The moment that the knife was pushed through the cartilages of the ribs, bloody serum gushed out in great quantities, and the lungs were protruded through the incision of the left side with much force. This was occasioned by the pressure of the diaphragm upon the thoracic viscera: it seemed indeed ready to give way every minute, and deluge us with the abdominal collection of water. After sponging out a considerable quantity of water, the lung of the left side was found healthy; that of the left, hepatized, and also anasarcous: it was firmly adherent to the pleura costalis in all places. The pericardium was tense, and contained a few ounces of serum. The heart was considerably and equally enlarged; at least one-third more than its natural volume. It appeared to be rather active than passive enlargement. The auriculo-ventricular opening of the left heart was also very large, but the margins of the valves presented cartilage and incipient ossification. The aorta was enlarged, but the semilunar valves were sound. The origins of the two coronary arteries were larger than the Reporters ever remember to have seen; they would nearly admit the tip of the little finger. These arteries were slit open, and some of their branches appeared as large as the coronary arteries usually are found.

It is not improbable that the liver was diseased; but the ascites precluded the idea of examination.

The great peculiarity in this case is the remarkable phænomenon of non-pulsation of any of the tangible arteries, except the carotid, for so long a space of time, apparently without much inconvenience. The temporal artery was turgid and very distinctly visible. If any pulsation existed, it must have been easily felt. Are we to suppose, that there was no *motion* of the blood at this time through the vessels? or is it not more probable that the motion was so *languid,* in consequence of the embarrassed situation of the heart, that the phænomenon of pulsation was imperceptible, as in the veins, where the current is known to be so slow? When the bowels were set in motion, and the urinary secretion increased, the pulsations returned, and the heart obtained a momentary freedom of action. The drain from the legs procrastinated the fatal blow; and thoracic effusion, in its usual sudden and silent manner, snapped the thread of life without a moment's notice.

